# Trends and Disparities in Obesity‐Related Mortality Among U.S. Adults: A CDC WONDER Analysis (1968–2025)

**DOI:** 10.1002/osp4.70146

**Published:** 2026-04-14

**Authors:** Faizan Ahmed, Husnain Ahmed, Ayesha Ishtiaq, Najam Gohar, Anika Goel, Abdul Waheed, Tehmasp Rehman Mirza, Haris Bin Tahir, Fatima Saleemi, Tallal Mushtaq Hashmi, Ramsha Ali, Hritvik Jain, Mushood Ahmed, Farah Deshmukh, Mohamed Bakr, Swapnil Patel, Mohammad Hossain, Ameer Haider Cheema

**Affiliations:** ^1^ Jersey Shore University Medical Centre Neptune New Jersey USA; ^2^ Shalamar Medical & Dental College Lahore Pakistan; ^3^ Shaheed Zulfiqar Ali Bhutto Medical University Islamabad Pakistan; ^4^ Ameer‐ud‐Din Medical College Lahore Pakistan; ^5^ Kakatiya Medical College Warangal India; ^6^ Lahore General Hospital Lahore Pakistan; ^7^ Rawalpindi Medical University Rawalpindi Pakistan; ^8^ People's University of Medical and Health Sciences Nawabshah Pakistan; ^9^ All India Institute of Medical Sciences (AIIMS) Jodhpur India; ^10^ University of Texas Southwestern Medical Center Dallas Texas USA

**Keywords:** CDC WONDER, Epidemiology, mortality, obesity

## Abstract

**Context:**

Obesity‐related mortality in the United States has increased substantially over recent decades, yet long‐term national trends and demographic disparities remain incompletely characterized.

**Objective:**

To evaluate nationwide trends in obesity‐related mortality among U.S. adults and identify disparities by age, sex, race, and geographic region from *1968 to 2025*.

**Methods:**

We performed a retrospective analysis using CDC WONDER mortality data from 1968 to 2025 (nearly six decades). Adults aged ≥ 25 years with obesity identified using International Classification of Diseases (ICD) codes listed as an underlying or contributing cause of death were included. Age‐adjusted mortality rates (AAMRs) per 100,000 population were calculated using the 2000 U.S. standard population, and temporal trends were evaluated using Joinpoint regression to estimate annual percent change (APC) and average annual percent change (AAPC).

**Results:**

From 1968 to 2025, a total of 211,479 obesity‐related deaths were recorded among U.S. adults aged ≥ 25 years. AAMRs increased from 1.15 to 3.32 per 100,000, peaking in 2021 (4.54 per 100,000). Mortality rates were consistently higher among males, older adults, Black populations, and residents of the Southern United States, while younger adults (25–44 years) experienced the most rapid rise in mortality.

**Conclusion:**

Obesity‐related mortality in the United States has more than tripled over nearly six decades, with substantial demographic and geographic disparities. These findings highlight the urgent need for targeted, culturally tailored, and age‐specific public health interventions to address the growing burden of obesity.

## Graphical abstract

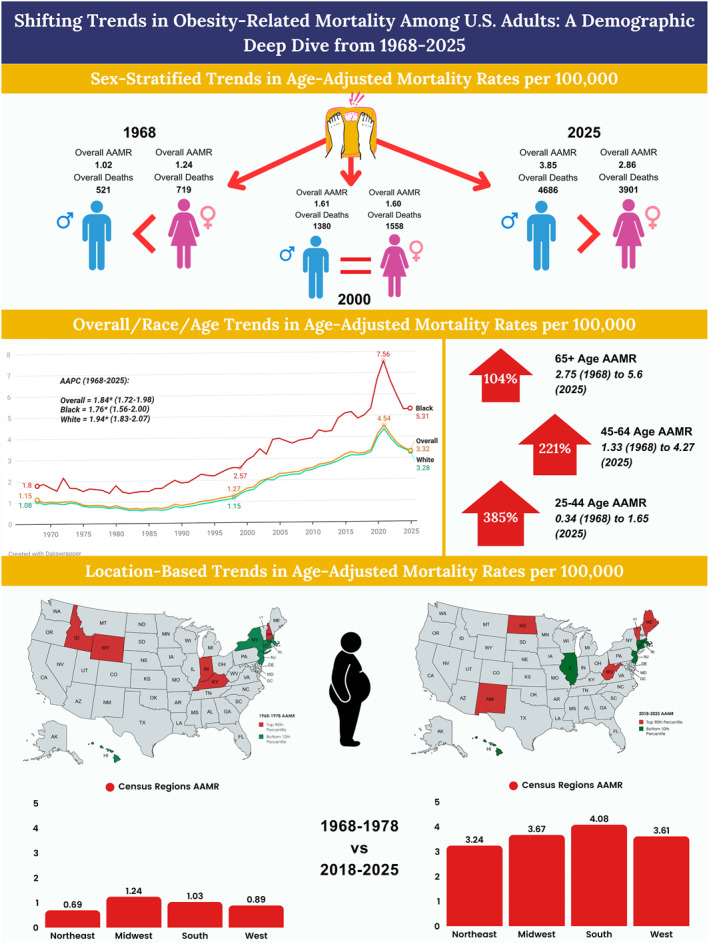

## 1 Introduction

Obesity remains one of the most significant public health challenges in the United States, affecting more than 40% of adults and over 100 million individuals nationwide, with severe obesity present in nearly 10% of the population [1]. Over the past several decades, the prevalence of obesity has risen substantially, increasing from approximately 30% in 2000 to more than 40% in recent national estimates, with marked disparities across racial, socioeconomic, and geographic groups [1, 2]. This growing epidemic has contributed to escalating rates of cardiovascular disease, type 2 diabetes, multiple malignancies, and premature mortality, placing a considerable burden on healthcare systems and public health infrastructure [3, 4].

Beyond cardiometabolic disease, obesity is increasingly recognized as a multisystem disorder. Excess adiposity has been identified as one of the strongest modifiable risk factors for knee osteoarthritis, significantly contributing to disability and reduced quality of life among aging populations [5]. Furthermore, maternal obesity has been associated with increased risks of congenital anomalies and adverse perinatal outcomes, underscoring the intergenerational health implications of the obesity epidemic [6].

Recent years have also witnessed rapid advances in the clinical management of obesity. Pharmacologic therapies targeting metabolic pathways, particularly glucagon‐like peptide‐1 receptor agonists (GLP‐1RAs) such as semaglutide and related agents, have demonstrated substantial weight‐loss efficacy and cardiometabolic benefits in randomized clinical trials [7, 8]. However, emerging real‐world evidence suggests that treatment effectiveness may vary outside controlled trial settings due to factors such as medication adherence, dosing variability, and treatment discontinuation, which may affect long‐term outcomes and population‐level impact [9].

In parallel, increasing attention has been directed toward earlier interventions in the life course. Pharmacologic therapies including GLP‐1RAs are now being explored for the management of pediatric obesity, alongside digital and behavioral strategies aimed at modifying metabolic trajectories early in life and preventing long‐term complications [10, 11]. These developments reflect a growing recognition of obesity as a heterogeneous and chronic disease influenced by genetic, metabolic, environmental, and behavioral determinants [12].

Despite expanding knowledge of obesity pathophysiology and the emergence of novel therapeutic options, relatively little research has examined how these evolving clinical and epidemiologic dynamics translate into long‐term mortality patterns at the population level. Longitudinal analyses are particularly important for understanding how demographic factors such as age, sex, race, and geographic region shape obesity‐associated mortality over time.

To address this gap, this study analyzes 58 years of national mortality data from the CDC WONDER database (1968–2025) to evaluate temporal trends in obesity‐associated mortality among U.S. adults. By examining these patterns across demographic strata, this study provides a comprehensive population‐level perspective on the evolving mortality burden of obesity and offers important context for ongoing clinical and public health interventions.

## 2 Methods

### 2.1 Study Design

The study analyzed mortality data related to obesity obtained from death certificate information through the CDC Wide‐Ranging Online Data for Epidemiologic Research (CDC WONDER) system [13]. CDC WONDER is a publicly available database commonly used to examine age‐adjusted mortality rates (AAMRs) and trends in disease‐related mortality in the United States. The database allows stratification by demographic and geographic variables, including sex, race/ethnicity, 2013 urbanization classification, and U.S. census regions, which facilitates the identification of high‐risk populations.

Mortality data from 1968 to 2025 were extracted using International Classification of Diseases (ICD) codes for obesity, including ICD‐8 code 277 (1968–1978), ICD‐9 code 278 (1979–1998), and ICD‐10 code E66 (1999–2025) [14]. The dataset included cause‐of‐death information from death certificates across all 50 states, focusing on deaths where obesity was listed as the underlying or a contributing cause of death. The study included adults aged 25 years and older at the time of death.

Institutional Review Board (IRB) approval was not required because the study used de‐identified, publicly available data from the CDC WONDER database, posing minimal risk to confidentiality or privacy. To ensure transparency and methodological rigor, the study followed the Strengthening the Reporting of Observational Studies in Epidemiology (STROBE) guidelines [15]. A completed STROBE checklist is provided in the Supporting Information S1: Table S1.

### 2.2 Data Extraction

Data were extracted for several variables, including population size, year of death, geographic region, sociodemographic characteristics, and state‐level information. Demographic variables included sex, age group, and race/ethnicity. The location of death was categorized as medical facility (outpatient, emergency department, inpatient, death on arrival, or unknown status), home, hospice, or nursing home/palliative care facility.

For the entire study period, racial categories included White and Black or African American. Additional racial/ethnic groups, including American Indian or Alaska Native and Asian or Pacific Islander, were available from 1999 to 2025 following the adoption of ICD‐10 coding in national mortality datasets. These variables were obtained from death certificates and are commonly used in CDC WONDER–based mortality analyses. Geographic regions were defined according to the U.S. Census Bureau classifications, including the Northeast, Midwest, South, and West [16].

### 2.3 Statistical Analysis

To analyze national trends in obesity‐related mortality, crude mortality rates per 100,000 population were calculated by dividing the number of obesity‐related deaths by the total U.S. population for each year. Mortality rates were examined across different age groups with 95% confidence intervals (CIs).

Age‐adjusted mortality rates (AAMRs) were calculated by applying age‐specific mortality rates to the 2000 U.S. standard population, allowing comparisons across populations and time periods. AAMRs were stratified by sex, race, census region, and state.

Temporal trends were evaluated using the Joinpoint Regression Program (version 5.2.0; National Cancer Institute). This method estimates Annual Percentage Changes (APCs) and Average Annual Percentage Changes (AAPCs) in AAMRs using log‐linear regression models. Joinpoint models began with the simplest model permitted by the data and sequentially added joinpoints up to a specified maximum. APCs were considered statistically significant when the slope differed from zero using two‐tailed *t*‐tests with *p* < 0.05.

Both crude and age‐adjusted mortality rates per 100,000 individuals from 1968 to 2025 were evaluated to assess regional trends in obesity‐related mortality. AAMRs standardized to the 2000 U.S. population were obtained from the CDC WONDER database, along with 95% confidence intervals, standard errors, and population estimates. Because the study used de‐identified, publicly available mortality data, institutional review board approval and informed consent were not required.

## 3 Results

### 3.1 Overall

**FIGURE 1:**
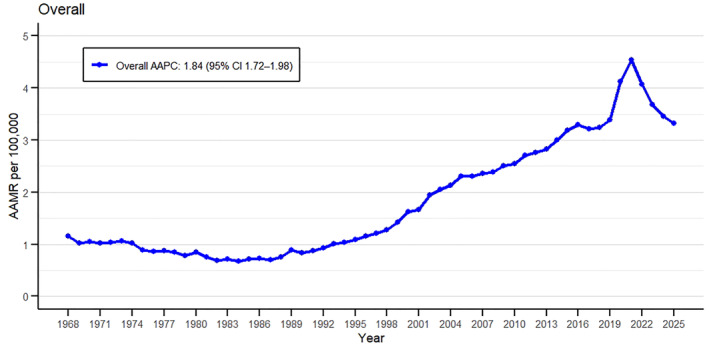
Overall trends in obesity‐related mortality in U.S. adults, 1968–2025, showing age‐adjusted mortality rates (AAMRs) per 100,000 population from the CDC WONDER database.

Between 1968 and 2025, a total of 211,479 obesity‐related deaths were recorded among U.S. adults aged ≥ 25 years (Supporting Information S1: Table S2). The age‐adjusted mortality rates (AAMR) ranged between 1.15 per 100,000 (95% CI 1.08–1.22) in 1968 and 3.32 per 100,000 (95% CI 3.25–3.40) in 2025 (Figure 1, Supporting Information S1: Table S3). The peak AAMR per 100,000 was reported in 2021 at 4.54 (95% CI 4.45–4.62) with 11,172 deaths. Despite the increasing number of deaths in successive years, the mortality rates had demonstrated major variations with the average annual percentage change (AAPC) of 1.84 (95% CI 1.72–1.98; *p* < 0.001). The extreme upward spike in the mortality rates was observed from 2018 to 2021 with the annual percentage change (APC) of 10.11 (95% CI 7.68–11.55; *p* < 0.001) while the major downward spike was observed from 2021 to 2025 with APC −7.75 (95% CI −9.12 to −6.53; *p* < 0.001) (Supporting Information S1: Table S4, S5).

### 3.2 Sex Stratification

**FIGURE 2:**
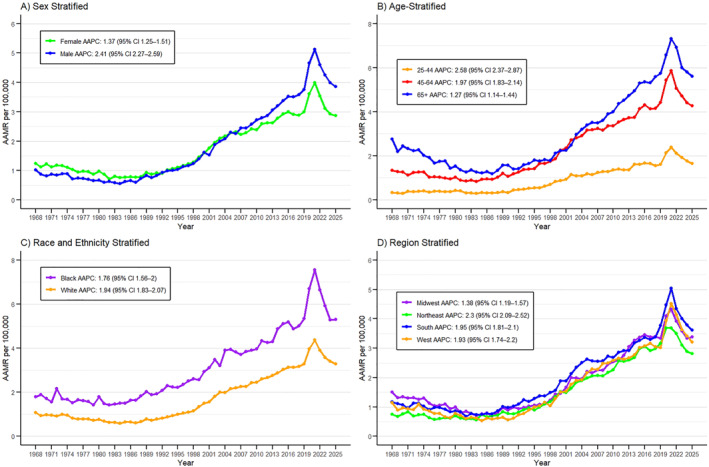
Trends in obesity‐related mortality among U.S. adults aged ≥ 25 years, 1968–2025, stratified by demographic and geographic characteristics. (A) Sex‐stratified trends (B) Age‐group trends (25–44, 45–64, ≥ 65 years) (C) Race‐stratified trends (Black and White populations) (D) U.S. Census region–stratified trends (Northeast, Midwest, South, West). Age‐adjusted mortality rates (AAMRs) are expressed per 100,000 population and were derived from the CDC WONDER database.

Segregation of the database by sex from 1968 to 2025 revealed similar statistics for both the males and females having comparable AAMRs with major variations. The female population had a higher number of mortality recorded i.e. 106,833 deaths and the male population had a total of 104,646 deaths from 1968 to 2025. In females, the age‐adjusted mortality rates (AAMRs) per 100,000 extended from 1.24 (95% CI 1.15–1.33) in 1968 to 2.86 (95% CI 2.77–2.96) in 2025, whereas in males it ranged from 1.02 (95% CI 0.93–1.11) to 3.85 (95% CI 3.74–3.96) over the same period (Figure 2A). Sex‐specific AAMRs are provided in Supporting Information S1: Table S3. The highest AAMR per 100,000 in both the sexes was recorded in 2021 that is 3.98 (95% CI 3.87–4.09) in females and 5.12 (95% CI 4.99–5.26) in males.

Despite some variations, Joinpoint analysis revealed rising mortality trajectories for both sexes, with AAPC of 1.37 (95% CI 1.25–1.51; *p* < 0.001) in females, with a major upward spike observed from 1997 to 2003 (APC 9.97; 95% CI 7.71–14.51; *p* < 0.001), and AAPC of 2.41 (95% CI 2.27–2.59; *p* < 0.001) in males, with a major upward spike from 2018 to 2021 (APC 11.14; 95% CI 8.59–12.67; *p* < 0.001). Both sexes demonstrated a peak downward trend from 2021 to 2025, with females showing APC −8.16 (95% CI −9.78 to −6.70; *p* < 0.001) and males APC −6.98 (95% CI −8.36 to −5.72; *p* < 0.001) (Supporting Information S1: Table S4, S5).

### 3.3 Age Group Stratification

The data for the mortality trends for obesity were divided into the three main age groups: young adults (25–44 years), middle age (45–64 years), and older adults (65+). The overall highest number of deaths over the study period was in the middle‐aged adults (98,626), followed by older adults (72,317) and young adults (40,536). All the age groups showed an increase in the mortality trends in successive years, with the elder group (65+) consistently demonstrating the highest most mortality rates (Figure 2B). The AAMR in elders progressed from 2.75 per 100,000 in 1968 to 5.60 per 100,000 in 2025. The highest AAMR per 100,000 for this age group was recorded in year 2022 at 6.92 (95% CI 6.71–7.14). On the other hand, AAMR in the middle age group rose from 1.33 per 100,000 in 1968 to 4.27 per 100,000 in 2025. The highest AAMR per 100,000 for this age group was recorded in year 2021 at 5.85 (95% CI 5.68–6.02). Lastly, AAMR in young adults rose from 0.34 per 100,000 in 1968 to 1.65 per 100,000 in 2025. The highest AAMR per 100,000 for this age group was recorded in year 2021 at 2.38 (95% CI 2.28–2.49) (Supporting Information S1: Table S6).

Joinpoint analysis demonstrated broadly similar temporal patterns across the three age groups from 1968 to 2025, with notable increases between 2018 and 2021 followed by declines during 2021–2025. The corresponding APCs were 12.46 and −8.55 for young adults, 10.05 and −7.84 for middle‐aged adults, and 8.35 and −6.52 for older adults (all *p* < 0.001).

Overall, from 1968 to 2025, the AAPCs were 2.58 (95% CI 2.37–2.87; *p* < 0.001) for young adults, 1.97 (95% CI 1.83–2.14; *p* < 0.001) for middle‐aged adults, and 1.27 (95% CI 1.14–1.44; *p* < 0.001) for older adults.

### 3.4 Race Stratification

The database considering mortality trends for Obesity in 1968–2025 was segregated into two main categories: Black or African American and White individuals. A total of 39,944 deaths were reported in the black population and 167,572 deaths in the white population. White AAMRs per 100,000 extended from 1.08 (95% CI 1.02–1.15) in 1968 to 3.28 (95% CI 3.19–3.36) in 2025, whereas in Black populations it ranged from 1.80 (95% CI 1.53–2.07) to 5.31 (95% CI 5.05–5.58) over the same period (Figure 2C). The highest AAMR per 100,000 was recorded in 2021 at 4.37 (95% CI 4.27–4.46) for White and 7.56 (95% CI 7.24–7.88) for Black (Supporting Information S1: Table S7).

Joinpoint analysis indicated major variations in mortality trends over time, with AAPC values of 1.76 (95% CI 1.56–2.00; *p* < 0.001) for Blacks and 1.94 (95% CI 1.83–2.07) (*p* < 0.001) for Whites. A notable upward spike was seen in 1997–and a downward trend was seen from 2021 to 2025 for Whites at APC 10.84 (95% CI 8.67–15.12; *p* < 0.001) and −6.80 (95% CI −8.15 to −5.54; *p* < 0.001), respectively. Similarly, Blacks exhibited significant rise from 2018 to 2021 & decrease from 2021–2025 at APC 12.86 (95% CI 8.56–15.34; *p* < 0.001) and −9.05 (95% CI −11.08 to −7.19; *p* < 0.001) respectively.

**FIGURE 3:**
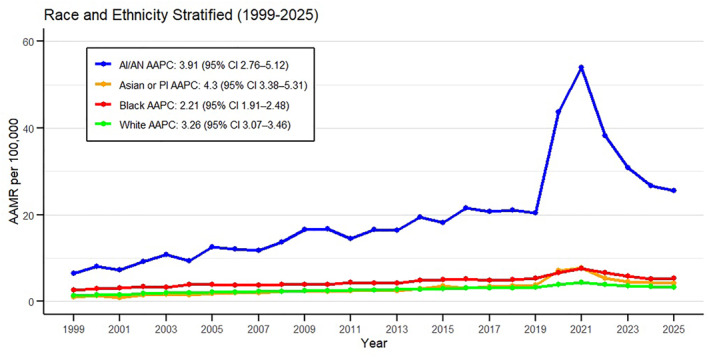
Race‐stratified trends in obesity‐related mortality in U.S. adults, 1999–2025, showing age‐adjusted mortality rates (AAMRs) per 100,000 population from the CDC WONDER database.

A further analysis was conducted to explore the trends of American Indian or Alaskan Native (AI/AN) and Asian or Pacific Islander (A/PI) populations from 1999 to 2025, as limited by the database. AI/AN AAMRs extended from 6.5 (95% CI 5.01–8.31) per 100,000 in 1999 to 25.59 (95% CI 23.74–27.55) in 2025 with the highest AAMR in 2021 at 53.99 (95% CI 51.17–56.95). A/PI AAMRs also rose from 1.00 (95% CI 0.76–1.29) in 1999 to 4.24 (95% CI 3.93–4.56) in 2025 with highest AAMR in 2021 at 7.77 (95% CI 7.32–8.23) paralleling other races (Figure 3, Supporting Information S1: Table S8). Temporal analyses revealed a steeper rise in these races than Black and Whites over the same period (1999–2025), with AAPC values of 4.30 (95% CI 3.38–5.31; *p* < 0.001) for A/P1, 3.91 (95% CI 2.76–5.12; *p* < 0.001) for AI/AN; while, 3.26 (95% CI 3.07–3.46; *p* < 0.001) for Whites and 2.21 (95% CI 1.91–2.48; *p* < 0.001) for Blacks. The notable upward spike was seen in 2018–2021 & downward trend was seen from 2021 to 2025 for both races. AI/AN rise was of APC 32.71 (95% CI 17.05–40.73; *p* < 0.001) with a decline of APC −18.37 (95% CI −24.96 to −13.80; *p* < 0.001). A/PI rise was of APC 25.89 (95% CI 14.63–32.54; *p* < 0.001) and fall of APC −15.69 (95% CI −22.02 to −11.30; *p* < 0.001) (Supporting Information S1: Table S4, S5).

### 3.5 Geographical Region Stratification (Census & States)

The database considering mortality trends for obesity was segregated into four geographical areas that is Northeast, Midwest, South and West. Regionally, the highest number of deaths in the United States from 1968–2025 was observed in the South (83789), followed by the Midwest (48183), West (44034) and Northeast (35473). Across the study period, all U.S. regions demonstrated increasing age‐adjusted mortality rates (AAMRs). In the Northeast, AAMRs increased from 0.75 (95% CI 0.65–0.85) per 100,000 in 1968 to 2.81 (95% CI 2.65–2.97) in 2025. In the Midwest, AAMRs rose from 1.50 (95% CI 1.36–1.64) to 3.38 (95% CI 3.22–3.55). Similarly, AAMRs in the South increased from 1.17 (95% CI 1.05–1.29) in 1968 to 3.62 (95% CI 3.50–3.74) in 2025, while those in the West rose from 1.14 (95% CI 0.98–1.31) to 3.20 (95% CI 3.06–3.35) over the same period (Figure 2D). The highest AAMR per 100,000 was recorded in 2021 at 3.69 (95% CI 3.51–3.87) for Northeast; 4.33 (95% CI 4.14–4.51) for Midwest; 5.04 (95% CI 4.90–5.19) for South; and 4.52 (95% CI 4.35–4.70) for West (Supporting Information S1: Table S9).

Joinpoint analysis showed major variations among different regions with the major upward spikes as follows: 1995–2003 in Northeast at APC 8.22 (95% CI 3.20–14.42; *p* = 0.007), 1997–2002 in Midwest at APC 10.56 (95% CI 6.43–16.62); *p* = 0.005), 2018–2021 in South at APC 12.76 (95% CI 10.10–14.33); *p* < 0.001), 2018–2021 in West at APC 12.90 (95% CI 7.72–15.31; *p* < 0.001).

In 2021–2025, all the regions demonstrated their peak downward spikes in such a way: Northeast at APC −6.43 (95% CI −9.35 to −4.11; *p* < 0.001), Midwest at APC −5.39 (95% CI −8.81 to −2.86; *p* < 0.001), South at APC −7.99 (95% CI −9.14 to −6.76; *p* < 0.001), West at APC −8.00 (95% CI −10.39 to −6.20; *p* < 0.001). Over the entire interval (1968–2025), AAPCs were 2.30 (95% CI 2.09–2.52; *p* < 0.001) in the Northeast, 1.38 (95% CI 1.19–1.57; *p* < 0.001) in the Midwest, 1.95 (95% CI 1.81–2.10; *p* < 0.001) in the South, and 1.93 (95% CI 1.74–2.20; *p* < 0.001) in the West (Supporting Information S1: Table S4).

**FIGURE 4:**
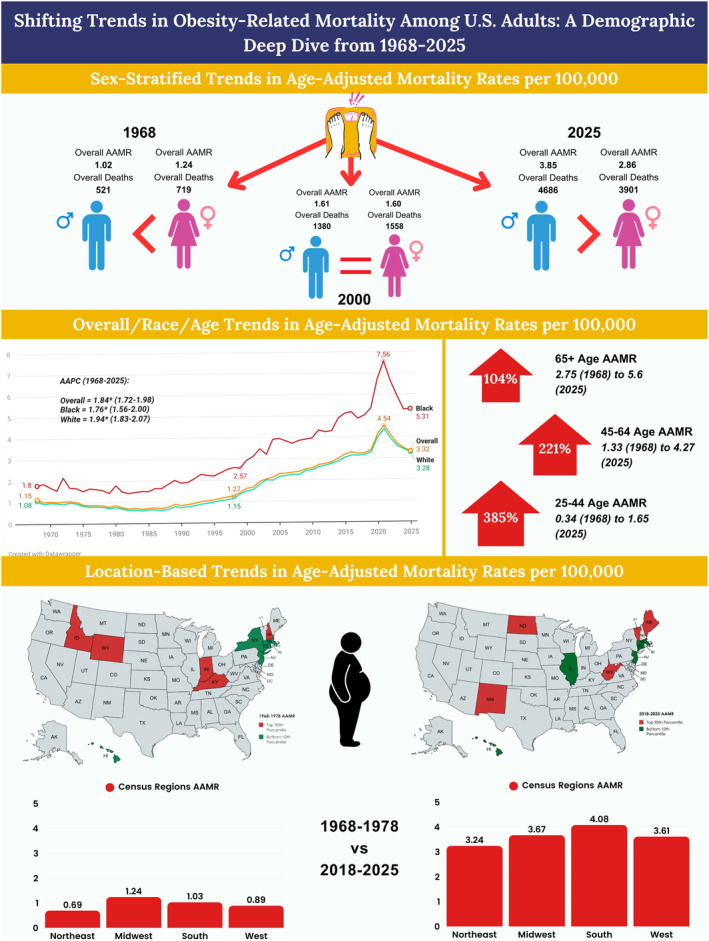
Central illustration of obesity‐related mortality trends in U.S. adults, 1968–2025.

At the state level, New Hampshire, Kansas, Wisconsin, Idaho and Indiana stayed at or above the 90th percentile in average Obesity related mortality rates from 1968 to 1978, whereas New York, New Jersey, Connecticut, Massachusetts and Hawaii remained below the 10th percentile (Supporting Information S1: Table S10). On the other hand, recently from 2018 to 2025, West Virginia, New Mexico, North Dakota, Vermont and Maine stayed at or above the 90th percentile, whereas New Jersey, Illinois, Connecticut, Massachusetts and Hawaii remained below the 10th percentile (Supporting Information S1: Table S11). The regions in the bottom 10th percentile remain largely unchanged. A central illustration provides an integrated overview of sex, race, age and region‐stratified trends in obesity‐related mortality across the period (Figure 4).

## 4 Discussion

This nearly six‐decade, population‐level analysis indicates that obesity has changed from a comorbid health issue to a significant contributor to mortality in the United States. Indeed, age‐adjusted mortality rates (AAMR) linked to obesity increased from 1968 to 2025 by nearly threefold, with AAMR reaching a peak of 4.54 per 100,000 in 2021. The increase in AAMR (APC = 10.11) between 2018 and 2021 in our data set most likely signifies the interaction of chronic disease and acute viral infection, especially during the COVID‐19 pandemic. Obesity was identified as an independent predictor of hospitalization, ICU admission for severe acute respiratory syndrome (SARS), and COVID‐19‐related mortality [17, 18]. Multiple analyses have shown that > 30% of hospitalized patients with COVID‐19 had obesity, contributing to a substantial proportion of excess mortality observed during the pandemic [19].

Marked variations in mortality were also observed over time, with a notable decline between 2021 and 2025 (APC = −7.75), most likely reflecting a post‐pandemic correction to excess mortality or lag effects of deaths being reported [19]. While such episodic reversals may occur, they do not appear to offset the long‐term upward trend (AAPC = 1.84), which remains statistically significant and clinically relevant.

Trends have also been analyzed by sex, where males and females experienced similar AAMRs until the late 1990s when mortality among males began to accelerate upward, peaking at 5.12 per 100,000 in 2021. The changes observed may have biological determinants such as a higher deposition of visceral fat in men. There is also evidence suggesting that men are less likely to participate in preventive care or obesity treatment [20, 21]. On the contrary, women contributed a greater number of deaths even though females generally have a longer life expectancy and higher health‐seeking behaviors, which may delay but not necessarily prevent death from obesity‐related conditions [22].

The age‐stratified results found that the highest number of deaths occurred in middle‐aged adults (45–64 years), but the highest age‐adjusted mortality rates were observed in older adults (65+). Middle‐aged obesity being a strong predictor of cardiovascular, oncologic and all‐cause mortality in later life has been demonstrated by the Nurses' Health Study [23]. As a concerning additional note, young adults (25–44 years) experienced the greatest increase in AAMR (AAPC = 2.58). Joinpoint analysis further demonstrated a rapid increase in mortality between 2018 and 2021 across all age groups. A recent study estimated that if these trends persist, over 55% of today's generation will have obesity by the age of 35 [24]. This early life onset of obesity not only shortens life expectancy but also increases years lived with disability (YLD), which has become increasingly important in discussions of epidemiological transition [25].

Racial disparities were both alarming and pronounced. African Americans experienced higher AAMRs throughout the study period and peaked at 7.56 per 100,000 in 2021, which was nearly double that of Whites. Consistent with overall patterns of health inequities, disparities driven by structural racism, limited healthcare access, food insecurity and chronic stressors may contribute to the increasing prevalence of obesity and negatively influence health outcomes [26]. Additionally, compared to Whites, Black populations tend to have higher rates of comorbid conditions such as hypertension and type 2 diabetes, which elevate the risk of mortality among individuals with obesity [27].

Additional analyses including American Indian or Alaska Native (AI/AN) and Asian or Pacific Islander populations revealed substantial increases in obesity‐related mortality during the more recent study period. AI/AN populations demonstrated particularly high mortality rates, exceeding 25 per 100,000 in 2025 and peaking during the pandemic period. These findings are consistent with prior literature documenting disproportionately high burdens of cardiometabolic disease among Indigenous populations, which may reflect longstanding structural inequities in healthcare access, socioeconomic disadvantage and geographic barriers to care [28, 29]. Mortality rates among Asian or Pacific Islander populations also increased steadily over time, suggesting that obesity‐related mortality may be emerging as a growing concern in populations historically perceived to have lower cardiometabolic risk [30].

The geographical perspective further describes the intersection of social determinants with health outcomes. The South consistently reported the highest rates of mortality from obesity‐related causes. This aligns with the findings of associated reports and evidence from the Stroke Belt, where limited access to medical care, reduced physical activity and diets high in saturated fats are prevalent [31]. By the end of the study period, the South continued to demonstrate higher obesity‐related mortality compared with other regions, reinforcing regional clustering of cardiometabolic deaths associated with obesity.

States like West Virginia and Louisiana have historically been markers of overweight and consistently remained in the higher percentiles for obesity‐related death rates [32]. At the other hand, states like Massachusetts and Hawaii, which made large investments in public health infrastructure and education, remained among the lowest percentiles. These states present natural laboratories for evaluating state‐level interventions and policies, such as soda taxes, urban walkability programs and community‐based physical activity initiatives [33].

This study has several advantages from a methodological standpoint. First, nearly six decades of national mortality data represent one of the most comprehensive longitudinal examinations of obesity‐attributable mortality in the United States. Second, Joinpoint regression allows accurate identification of the time points at which significant changes in mortality trends occur. Third, the study provided robust stratification by age, gender, race and region, offering detailed insights into high‐risk subpopulations that are often under‐represented in national analyses. However, several limitations should be acknowledged. Death certificates may underreport obesity as a contributing cause of death due to stigma, misclassification and competing comorbidities such as diabetes or cardiovascular disease [34, 35]. CDC WONDER data are observational and therefore do not allow causal inference. Additionally, obesity risk modifiers such as Hispanic ethnicity, education level and socioeconomic status were not included in this analysis.

The clinical implications uncovered are substantial. The persistent rise in mortality due to obesity, particularly among younger and middle‐aged adults, suggests that current preventive strategies may be insufficient to address the expanding obesity epidemic. Preventive interventions remain essential and may require integrated public health strategies including nutritional programs, early metabolic screening, regulation of processed foods and sugar‐sweetened beverages, and improved access to obesity treatment. Furthermore, the observed racial and geographic variations highlight the importance of culturally appropriate public health interventions and equitable access to obesity treatment modalities such as pharmacotherapy and bariatric surgery [36, 37].

Moreover, future research should focus on identifying longitudinal relationships between obesity and cause‐specific mortality (such as ischemic heart disease, cancers and respiratory diseases) using multiple‐cause‐of‐death data. Greater emphasis on socio‐demographic, biological, behavioral and environmental exposures may further improve risk prediction and inform public health planning [38]. Geographic analytical techniques may also help identify regional opportunities for intervention. Additionally, evaluating population‐level mortality changes associated with newer anti‐obesity pharmacologic therapies, including GLP‐1 receptor agonists, may be important in future studies. Furthermore, mortality data for the most recent years (2024–2025) in the CDC WONDER database may represent provisional estimates and could be subject to revision as additional death certificate data become finalized.

## 5 Conclusion

In conclusion, obesity‐related mortality in the United States has increased substantially over nearly six decades, with persistent disparities across age, sex, race and geographic regions. Although mortality rates peaked during the COVID‐19 pandemic and have shown a modest decline in recent years, the overall long‐term trajectory remains upward. These findings highlight the urgent need for targeted, equitable and population‐level interventions to address the growing burden of obesity‐related mortality.

## Funding

The authors have nothing to report.

## Ethics Statement

This study used de‐identified, publicly available mortality data from the CDC WONDER database and did not involve direct human participants or animal subjects.

## Consent

Institutional review board approval and informed consent were therefore not required.

## Conflicts of Interest

The authors declare no conflicts of interest related to this work. All authors have completed the ICMJE disclosure form and have reviewed and approved the final manuscript.

## Supporting information


Supporting Information S1

## Data Availability

All data used in this study are publicly available through the CDC WONDER online database and are also presented in the manuscript and its Supporting Information S1.
