# Supplementary material for: Trends and Disparities in Obesity‐Related Mortality Among U.S. Adults: A CDC WONDER Analysis (1968–2025)
Source: Obes Sci Pract. 2026 Apr 14;12(2):e70146. doi: 10.1002/osp4.70146 (PMC13080113; doi:10.1002/osp4.70146)
Supplement: Supplementary file 1 — Supporting Information S1 [file OSP4-12-e70146-s001.docx]

**Supplementary Section**

**Supplementary Table 1.** STROBE Checklist for cohort studies

|  | | Item No | Recommendation | Page No |
| --- | --- | --- | --- | --- |
| **Title and abstract** | | 1 | (*a*) Indicate the study’s design with a commonly used term in the title or the abstract | Page no 1,2 |
|  |  |  | (*b*) Provide in the abstract an informative and balanced summary of what was done and what was found |  |
| Introduction | | | | |
| Background/rationale | | 2 | Explain the scientific background and rationale for the investigation being reported | Page 3 |
| Objectives | | 3 | State specific objectives, including any prespecified hypotheses | Page 3 |
| Methods | | | | |
| Study design | | 4 | Present key elements of study design early in the paper | Page 4 |
| Setting | | 5 | Describe the setting, locations, and relevant dates, including periods of recruitment, exposure, follow-up, and data collection | Page 4 |
| Participants | | 6 | (*a*) Give the eligibility criteria, and the sources and methods of selection of participants. Describe methods of follow-up | Page 4-5 |
|  |  |  | (*b*) For matched studies, give matching criteria and number of exposed and unexposed | Not applicable |
| Variables | | 7 | Clearly define all outcomes, exposures, predictors, potential confounders, and effect modifiers. Give diagnostic criteria, if applicable | Page 5-6 |
| Data sources/ measurement | | 8* | For each variable of interest, give sources of data and details of methods of assessment (measurement). Describe comparability of assessment methods if there is more than one group | Page 5-6 |
| Bias | | 9 | Describe any efforts to address potential sources of bias | Page 19 |
| Study size | | 10 | Explain how the study size was arrived at | Page 4 |
| Quantitative variables | | 11 | Explain how quantitative variables were handled in the analyses. If applicable, describe which groupings were chosen and why | Page 6 |
| Statistical methods | | 12 | (*a*) Describe all statistical methods, including those used to control for confounding | Page 6-7 |
|  |  |  | (*b*) Describe any methods used to examine subgroups and interactions | Page 7-14 |
|  |  |  | (*c*) Explain how missing data were addressed | Not relevant |
|  |  |  | (*d*) If applicable, explain how loss to follow-up was addressed | Not applicable |
|  |  |  | (*e*) Describe any sensitivity analyses | Page 19 |
| Results | | | |  |
| Participants | | 13 | (a) Report numbers of individuals at each stage of study—eg numbers potentially eligible, examined for eligibility, confirmed eligible, included in the study, completing follow-up, and analysed | Page 7-8 |
|  |  |  | (b) Give reasons for non-participation at each stage | Not applicable |
|  |  |  | (c) Consider use of a flow diagram | Not applicable |
| Descriptive data | | 14* | (a) Give characteristics of study participants (eg demographic, clinical, social) and information on exposures and potential confounders | Page 8-14 |
|  |  |  | (b) Indicate number of participants with missing data for each variable of interest | Not relevant |
|  |  |  | (c) Summarise follow-up time (eg, average and total amount) | Not applicable |
| Outcome data | | 15* | Report numbers of outcome events or summary measures over time | Page 7-14 |
| Main results | 16 | (*a*) Give unadjusted estimates and, if applicable, confounder-adjusted estimates and their precision (eg, 95% confidence interval). Make clear which confounders were adjusted for and why they were included | | Page 7-8 |
|  |  | (*b*) Report category boundaries when continuous variables were categorized | | Page 5-6 |
|  |  | (*c*) If relevant, consider translating estimates of relative risk into absolute risk for a meaningful time period | | Page 7 |
| Other analyses | 17 | Report other analyses done—eg analyses of subgroups and interactions, and sensitivity analyses | | Page 8-14 |
| Discussion | | | | |
| Key results | 18 | Summarise key results with reference to study objectives | | Page 15-17 |
| Limitations | 19 | Discuss limitations of the study, taking into account sources of potential bias or imprecision. Discuss both direction and magnitude of any potential bias | | Page 19 |
| Interpretation | 20 | Give a cautious overall interpretation of results considering objectives, limitations, multiplicity of analyses, results from similar studies, and other relevant evidence | | Page 19-21 |
| Generalisability | 21 | Discuss the generalisability (external validity) of the study results | | Page 21 |
| Other information | | | | |
| Funding | 22 | Give the source of funding and the role of the funders for the present study and, if applicable, for the original study on which the present article is based | | Page 1 |

| **Supplementary Table 2: Overall and Sex-, Race-, and Age-Stratified Deaths due to Obesity among Adults in the United States (1968–2025)** | | | | | | | | |
| --- | --- | --- | --- | --- | --- | --- | --- | --- |
| **Year** | **Overall** | **Women** | **Men** | **Blacks** | **Whites** | **Young and Middle-aged Adults (25-44)** | **Old Adults (45-64)** | **Elderly (65+)** |
| **1968** | 1240 | 719 | 521 | 179 | 1057 | 155 | 556 | 529 |
| **1969** | 1120 | 671 | 449 | 187 | 926 | 142 | 543 | 435 |
| **1970** | 1162 | 735 | 427 | 173 | 981 | 133 | 544 | 485 |
| **1971** | 1127 | 684 | 443 | 161 | 958 | 177 | 482 | 468 |
| **1972** | 1188 | 740 | 448 | 224 | 954 | 168 | 550 | 470 |
| **1973** | 1217 | 741 | 476 | 178 | 1033 | 185 | 558 | 474 |
| **1974** | 1195 | 715 | 480 | 185 | 1005 | 197 | 573 | 425 |
| **1975** | 1064 | 673 | 391 | 168 | 890 | 171 | 458 | 435 |
| **1976** | 1053 | 636 | 417 | 189 | 860 | 195 | 467 | 391 |
| **1977** | 1075 | 660 | 415 | 187 | 883 | 202 | 465 | 408 |
| **1978** | 1063 | 661 | 402 | 187 | 866 | 188 | 453 | 422 |
| **1979** | 997 | 612 | 385 | 179 | 816 | 211 | 428 | 358 |
| **1980** | 1099 | 701 | 398 | 220 | 877 | 242 | 472 | 385 |
| **1981** | 993 | 637 | 356 | 193 | 794 | 236 | 408 | 349 |
| **1982** | 919 | 536 | 383 | 178 | 738 | 191 | 390 | 338 |
| **1983** | 977 | 608 | 369 | 191 | 770 | 193 | 417 | 367 |
| **1984** | 934 | 581 | 353 | 198 | 724 | 190 | 389 | 355 |
| **1985** | 1015 | 604 | 411 | 208 | 803 | 226 | 438 | 351 |
| **1986** | 1031 | 606 | 425 | 228 | 798 | 216 | 438 | 377 |
| **1987** | 1009 | 608 | 401 | 224 | 781 | 227 | 429 | 353 |
| **1988** | 1117 | 624 | 493 | 261 | 849 | 242 | 470 | 405 |
| **1989** | 1326 | 757 | 569 | 292 | 1022 | 275 | 566 | 485 |
| **1990** | 1253 | 719 | 534 | 275 | 969 | 257 | 504 | 492 |
| **1991** | 1361 | 762 | 599 | 293 | 1053 | 348 | 566 | 447 |
| **1992** | 1454 | 770 | 684 | 328 | 1113 | 377 | 615 | 462 |
| **1993** | 1615 | 882 | 733 | 368 | 1235 | 393 | 693 | 529 |
| **1994** | 1699 | 935 | 764 | 375 | 1312 | 427 | 717 | 555 |
| **1995** | 1812 | 994 | 818 | 376 | 1420 | 451 | 746 | 615 |
| **1996** | 1949 | 1059 | 890 | 412 | 1516 | 450 | 896 | 603 |
| **1997** | 2082 | 1125 | 957 | 456 | 1606 | 528 | 921 | 633 |
| **1998** | 2227 | 1198 | 1029 | 489 | 1716 | 589 | 1017 | 621 |
| **1999** | 2544 | 1351 | 1193 | 493 | 2023 | 672 | 1126 | 746 |
| **2000** | 2938 | 1558 | 1380 | 575 | 2329 | 757 | 1384 | 797 |
| **2001** | 3076 | 1714 | 1362 | 627 | 2406 | 755 | 1527 | 794 |
| **2002** | 3613 | 1935 | 1678 | 716 | 2851 | 936 | 1798 | 879 |
| **2003** | 3944 | 2114 | 1830 | 681 | 3206 | 918 | 1960 | 1066 |
| **2004** | 4144 | 2225 | 1919 | 832 | 3260 | 903 | 2084 | 1157 |
| **2005** | 4569 | 2388 | 2181 | 869 | 3632 | 969 | 2362 | 1238 |
| **2006** | 4664 | 2477 | 2187 | 852 | 3736 | 928 | 2437 | 1299 |
| **2007** | 4784 | 2414 | 2370 | 865 | 3842 | 986 | 2490 | 1308 |
| **2008** | 4945 | 2530 | 2415 | 914 | 3942 | 1046 | 2501 | 1398 |
| **2009** | 5340 | 2724 | 2616 | 938 | 4284 | 1033 | 2762 | 1545 |
| **2010** | 5467 | 2701 | 2766 | 962 | 4388 | 1066 | 2796 | 1605 |
| **2011** | 5879 | 2984 | 2895 | 1082 | 4700 | 1101 | 2992 | 1786 |
| **2012** | 6115 | 3075 | 3040 | 1069 | 4940 | 1050 | 3124 | 1941 |
| **2013** | 6369 | 3141 | 3228 | 1108 | 5144 | 1099 | 3160 | 2110 |
| **2014** | 6800 | 3347 | 3453 | 1282 | 5385 | 1293 | 3230 | 2277 |
| **2015** | 7350 | 3608 | 3742 | 1380 | 5809 | 1284 | 3537 | 2529 |
| **2016** | 7648 | 3718 | 3930 | 1432 | 6067 | 1321 | 3701 | 2626 |
| **2017** | 7668 | 3677 | 3991 | 1388 | 6133 | 1342 | 3602 | 2724 |
| **2018** | 7834 | 3719 | 4115 | 1428 | 6249 | 1293 | 3612 | 2929 |
| **2019** | 8286 | 3933 | 4353 | 1554 | 6588 | 1334 | 3858 | 3094 |
| **2020** | 10103 | 4678 | 5425 | 1983 | 7890 | 1776 | 4655 | 3672 |
| **2021** | 11172 | 5156 | 6016 | 2198 | 8647 | 2001 | 5041 | 4130 |
| **2022** | 10179 | 4676 | 5503 | 1952 | 7929 | 1790 | 4352 | 4037 |
| **2023** | 9228 | 4135 | 5093 | 1772 | 7178 | 1647 | 3982 | 3599 |
| **2024** | 8860 | 4001 | 4859 | 1608 | 6992 | 1562 | 3758 | 3540 |
| **2025** | 8587 | 3901 | 4686 | 1622 | 6697 | 1492 | 3626 | 3469 |
| **Total** | 211479 | 106833 | 104646 | 39944 | 167572 | 40536 | 98626 | 72317 |

| **Supplementary Table 3: Overall and Sex-Stratified Obesity-Related Age-Adjusted Mortality Rates (AAMRs) per 100,000 in Adults Aged 25 and Older in the United States (1968–2025)** | | | | | | | | | |
| --- | --- | --- | --- | --- | --- | --- | --- | --- | --- |
| **Year** | **Overall** | | | **Women** | | | **Men** | | |
|  | **AAMR** | **Lower 95% CI** | **Upper 95% CI** | **AAMR** | **Lower 95% CI** | **Upper 95% CI** | **AAMR** | **Lower 95% CI** | **Upper 95% CI** |
| **1968** | 1.15 | 1.08 | 1.22 | 1.24 | 1.15 | 1.33 | 1.02 | 0.93 | 1.11 |
| **1969** | 1.02 | 0.95 | 1.08 | 1.12 | 1.04 | 1.21 | 0.87 | 0.79 | 0.96 |
| **1970** | 1.05 | 0.99 | 1.11 | 1.22 | 1.13 | 1.31 | 0.81 | 0.73 | 0.89 |
| **1971** | 1.02 | 0.96 | 1.08 | 1.12 | 1.04 | 1.21 | 0.87 | 0.78 | 0.95 |
| **1972** | 1.04 | 0.97 | 1.1 | 1.18 | 1.09 | 1.27 | 0.85 | 0.77 | 0.93 |
| **1973** | 1.06 | 1 | 1.12 | 1.17 | 1.09 | 1.26 | 0.89 | 0.8 | 0.97 |
| **1974** | 1.02 | 0.96 | 1.07 | 1.11 | 1.03 | 1.19 | 0.89 | 0.81 | 0.97 |
| **1975** | 0.89 | 0.84 | 0.95 | 1.03 | 0.95 | 1.11 | 0.72 | 0.64 | 0.79 |
| **1976** | 0.86 | 0.81 | 0.92 | 0.95 | 0.87 | 1.02 | 0.74 | 0.67 | 0.81 |
| **1977** | 0.87 | 0.82 | 0.92 | 0.97 | 0.9 | 1.05 | 0.73 | 0.65 | 0.8 |
| **1978** | 0.85 | 0.8 | 0.9 | 0.96 | 0.88 | 1.03 | 0.7 | 0.63 | 0.77 |
| **1979** | 0.78 | 0.73 | 0.83 | 0.87 | 0.8 | 0.94 | 0.66 | 0.59 | 0.72 |
| **1980** | 0.85 | 0.8 | 0.9 | 0.98 | 0.91 | 1.06 | 0.67 | 0.6 | 0.74 |
| **1981** | 0.75 | 0.71 | 0.8 | 0.88 | 0.81 | 0.95 | 0.59 | 0.53 | 0.66 |
| **1982** | 0.69 | 0.64 | 0.73 | 0.72 | 0.66 | 0.78 | 0.63 | 0.57 | 0.7 |
| **1983** | 0.71 | 0.67 | 0.76 | 0.8 | 0.74 | 0.87 | 0.58 | 0.52 | 0.64 |
| **1984** | 0.67 | 0.63 | 0.72 | 0.76 | 0.7 | 0.83 | 0.56 | 0.5 | 0.61 |
| **1985** | 0.71 | 0.67 | 0.76 | 0.77 | 0.71 | 0.83 | 0.62 | 0.56 | 0.68 |
| **1986** | 0.73 | 0.68 | 0.77 | 0.78 | 0.71 | 0.84 | 0.65 | 0.59 | 0.71 |
| **1987** | 0.7 | 0.65 | 0.74 | 0.77 | 0.71 | 0.83 | 0.6 | 0.54 | 0.66 |
| **1988** | 0.76 | 0.72 | 0.81 | 0.78 | 0.72 | 0.84 | 0.73 | 0.66 | 0.8 |
| **1989** | 0.89 | 0.85 | 0.94 | 0.93 | 0.86 | 1 | 0.83 | 0.76 | 0.9 |
| **1990** | 0.83 | 0.79 | 0.88 | 0.87 | 0.8 | 0.93 | 0.76 | 0.69 | 0.82 |
| **1991** | 0.88 | 0.84 | 0.93 | 0.91 | 0.84 | 0.97 | 0.83 | 0.76 | 0.9 |
| **1992** | 0.93 | 0.88 | 0.98 | 0.9 | 0.84 | 0.97 | 0.93 | 0.86 | 1.01 |
| **1993** | 1.01 | 0.96 | 1.06 | 1.02 | 0.95 | 1.09 | 0.99 | 0.92 | 1.06 |
| **1994** | 1.04 | 0.99 | 1.09 | 1.06 | 0.99 | 1.13 | 1 | 0.92 | 1.07 |
| **1995** | 1.09 | 1.04 | 1.14 | 1.1 | 1.03 | 1.17 | 1.04 | 0.97 | 1.12 |
| **1996** | 1.16 | 1.11 | 1.21 | 1.17 | 1.1 | 1.24 | 1.13 | 1.05 | 1.2 |
| **1997** | 1.21 | 1.16 | 1.26 | 1.22 | 1.15 | 1.29 | 1.17 | 1.1 | 1.25 |
| **1998** | 1.27 | 1.22 | 1.32 | 1.28 | 1.21 | 1.35 | 1.24 | 1.16 | 1.31 |
| **1999** | 1.43 | 1.38 | 1.49 | 1.45 | 1.37 | 1.53 | 1.4 | 1.32 | 1.48 |
| **2000** | 1.62 | 1.56 | 1.68 | 1.6 | 1.52 | 1.68 | 1.61 | 1.52 | 1.69 |
| **2001** | 1.67 | 1.61 | 1.73 | 1.76 | 1.68 | 1.85 | 1.53 | 1.45 | 1.62 |
| **2002** | 1.94 | 1.88 | 2.01 | 1.95 | 1.86 | 2.03 | 1.87 | 1.78 | 1.96 |
| **2003** | 2.05 | 1.98 | 2.11 | 2.09 | 2 | 2.17 | 2 | 1.91 | 2.09 |
| **2004** | 2.13 | 2.06 | 2.19 | 2.17 | 2.07 | 2.26 | 2.08 | 1.99 | 2.17 |
| **2005** | 2.3 | 2.23 | 2.37 | 2.28 | 2.19 | 2.37 | 2.3 | 2.2 | 2.4 |
| **2006** | 2.31 | 2.24 | 2.37 | 2.32 | 2.23 | 2.41 | 2.26 | 2.16 | 2.36 |
| **2007** | 2.36 | 2.3 | 2.43 | 2.23 | 2.14 | 2.32 | 2.44 | 2.34 | 2.54 |
| **2008** | 2.39 | 2.32 | 2.46 | 2.29 | 2.2 | 2.38 | 2.45 | 2.35 | 2.55 |
| **2009** | 2.51 | 2.44 | 2.58 | 2.41 | 2.31 | 2.5 | 2.58 | 2.48 | 2.68 |
| **2010** | 2.55 | 2.48 | 2.62 | 2.39 | 2.3 | 2.48 | 2.72 | 2.62 | 2.82 |
| **2011** | 2.71 | 2.64 | 2.78 | 2.59 | 2.49 | 2.68 | 2.79 | 2.68 | 2.89 |
| **2012** | 2.76 | 2.69 | 2.83 | 2.62 | 2.52 | 2.71 | 2.86 | 2.75 | 2.96 |
| **2013** | 2.83 | 2.76 | 2.9 | 2.62 | 2.53 | 2.72 | 3.05 | 2.94 | 3.15 |
| **2014** | 3 | 2.92 | 3.07 | 2.78 | 2.68 | 2.88 | 3.2 | 3.09 | 3.31 |
| **2015** | 3.19 | 3.12 | 3.27 | 2.93 | 2.83 | 3.02 | 3.38 | 3.27 | 3.49 |
| **2016** | 3.29 | 3.22 | 3.37 | 3 | 2.9 | 3.1 | 3.52 | 3.41 | 3.64 |
| **2017** | 3.22 | 3.15 | 3.29 | 2.91 | 2.81 | 3.01 | 3.5 | 3.39 | 3.61 |
| **2018** | 3.24 | 3.16 | 3.31 | 2.88 | 2.79 | 2.98 | 3.58 | 3.47 | 3.69 |
| **2019** | 3.39 | 3.31 | 3.46 | 3 | 2.9 | 3.09 | 3.75 | 3.64 | 3.87 |
| **2020** | 4.13 | 4.05 | 4.21 | 3.61 | 3.5 | 3.72 | 4.66 | 4.53 | 4.79 |
| **2021** | 4.54 | 4.45 | 4.62 | 3.98 | 3.87 | 4.09 | 5.12 | 4.99 | 5.26 |
| **2022** | 4.07 | 3.99 | 4.15 | 3.53 | 3.42 | 3.63 | 4.6 | 4.48 | 4.73 |
| **2023** | 3.68 | 3.6 | 3.75 | 3.12 | 3.02 | 3.22 | 4.25 | 4.13 | 4.37 |
| **2024** | 3.46 | 3.39 | 3.53 | 2.93 | 2.83 | 3.02 | 3.98 | 3.87 | 4.1 |
| **2025** | 3.32 | 3.25 | 3.4 | 2.86 | 2.77 | 2.96 | 3.85 | 3.74 | 3.96 |
| AAMR = Age-Adjusted Mortality Rate; CI = Confidence Interval | | | | | | | | | |

| **Supplementary Table 4: Annual Percent Change (APC) of Obesity-Related Age-Adjusted Mortality Rates (AAMRs) per 100,000 in Adults Aged 25 and Older in the United States (1968–2025)** | | | | |
| --- | --- | --- | --- | --- |
| **Year Interval** | **APC** | **Lower 95% CI** | **Upper 95%**  **CI** | **P-value** |
| **Overall** | | | | |
| **1968-1985** | -3.03* | -3.78 | -2.43 | 0.0024 |
| **1985-1997** | 4.87* | 2.87 | 5.98 | 0.011998 |
| **1997-2003** | 9.79* | 7.66 | 14.32 | 0.0008 |
| **2003-2018** | 3.16* | 2.68 | 3.45 | 0.002 |
| **2018-2021** | 10.11* | 7.68 | 11.55 | < 0.000001 |
| **2021-2025** | -7.75* | -9.12 | -6.53 | < 0.000001 |
| **Women** | | | | |
| **1968-1986** | -2.89* | -3.58 | -2.34 | 0.002799 |
| **1986-1997** | 4.44* | 2.33 | 5.76 | 0.011198 |
| **1997-2003** | 9.97* | 7.71 | 14.51 | 0.0008 |
| **2003-2018** | 2.29* | 1.75 | 2.65 | 0.0008 |
| **2018-2021** | 8.97* | 5.96 | 10.56 | < 0.000001 |
| **2021-2025** | -8.16* | -9.78 | -6.7 | < 0.000001 |
| **Men** | | | | |
| **1968-1984** | -3.20* | -4.37 | -2.31 | 0.016397 |
| **1984-1997** | 5.71 | -1.41 | 6.92 | 0.066387 |
| **1997-2004** | 8.97* | 6.72 | 13.2 | 0.0008 |
| **2004-2018** | 3.84* | 3.18 | 4.24 | 0.0016 |
| **2018-2021** | 11.14* | 8.59 | 12.67 | 0.0008 |
| **2021-2025** | -6.98* | -8.36 | -5.72 | < 0.000001 |
| **Blacks or African Americans** | | | | |
| **1968-1984** | -1.53* | -2.91 | -0.35 | 0.011998 |
| **1984-2004** | 4.60* | 4.01 | 6.51 | 0.002 |
| **2004-2018** | 2.61* | 0.54 | 3.16 | 0.039192 |
| **2018-2021** | 12.86* | 8.56 | 15.34 | 0.0004 |
| **2021-2025** | -9.05* | -11.08 | -7.19 | < 0.000001 |
| **Whites** | | | | |
| **1968-1985** | -3.40* | -4.05 | -2.83 | 0.0008 |
| **1985-1997** | 5.04* | 3.34 | 6.15 | 0.003599 |
| **1997-2003** | 10.84* | 8.67 | 15.12 | < 0.000001 |
| **2003-2018** | 3.27* | 2.75 | 3.61 | 0.0008 |
| **2018-2025** | 9.49* | 6.88 | 10.89 | < 0.000001 |
| **American Indian or Alaska Native** | | | | |
| **1999-2018** | 5.18* | 2.84 | 7.04 | 0.0024 |
| **2018-2021** | 32.71* | 17.05 | 40.73 | < 0.000001 |
| **2021-2025** | -18.37* | -24.96 | -13.8 | < 0.000001 |
| **Asian or Pacific Islander** | | | | |
| **1999-2018** | 5.89* | 3.97 | 7.36 | 0.004399 |
| **2018-2021** | 25.89* | 14.63 | 32.54 | < 0.000001 |
| **2021-2025** | -15.69* | -22.02 | -11.3 | < 0.000001 |
| **Northeast** | | | | |
| **1968-1983** | -1.49* | -4.72 | -0.32 | 0.031594 |
| **1983-1995** | 3.85* | 0.24 | 6.39 | 0.040792 |
| **1995-2003** | 8.22* | 3.2 | 14.42 | 0.007199 |
| **2003-2021** | 3.97* | 3.51 | 4.65 | 0.004399 |
| **2021-2025** | -6.43* | -9.35 | -4.11 | < 0.000001 |
| **Midwest** | | | | |
| **1968-1985** | -3.98* | -5.27 | -3.08 | 0.008398 |
| **1985-1997** | 3.43 | -0.95 | 5.3 | 0.069986 |
| **1997-2002** | 10.56* | 6.43 | 16.62 | 0.005199 |
| **2002-2021** | 4.21* | 3.76 | 4.64 | 0.0008 |
| **2021-2025** | -5.39* | -8.81 | -2.86 | 0.0004 |
| **South** | | | | |
| **1968-1985** | -2.64* | -3.56 | -1.89 | 0.009998 |
| **1985-1999** | 5.67* | 0.11 | 6.53 | 0.04999 |
| **1999-2003** | 10.18* | 6.4 | 13.59 | 0.004399 |
| **2003-2018** | 2.48* | 2.03 | 2.82 | 0.0024 |
| **2018-2021** | 12.76* | 10.1 | 14.33 | < 0.000001 |
| **2021-2025** | -7.99* | -9.14 | -6.76 | < 0.000001 |
| **West** | | | | |
| **1968-1988** | -3.35* | -4.42 | -2.32 | < 0.000001 |
| **1988-2006** | 8.50* | 7.76 | 9.64 | < 0.000001 |
| **2006-2018** | 2.29* | 0.92 | 2.96 | 0.014797 |
| **2018-2021** | 12.90* | 7.72 | 15.31 | < 0.000001 |
| **2021-2025** | -8.00* | -10.39 | -6.2 | < 0.000001 |
| **Young and Middle-aged Adults (25-44)** | | | | |
| **1968-1988** | -0.78 | -1.81 | 0.26 | 0.131974 |
| **1988-2003** | 8.55* | 7.63 | 9.92 | 0.0008 |
| **2003-2018** | 2.60* | 1.5 | 3.21 | 0.005599 |
| **2018-2021** | 12.46* | 7.22 | 15.09 | < 0.000001 |
| **2021-2025** | -8.55* | -11.17 | -6.5 | < 0.000001 |
| **Old Adults (45-64)** | | | | |
| **1968-1984** | -2.87* | -3.88 | -2.06 | 0.0024 |
| **1984-1998** | 5.20* | 3.62 | 6.29 | 0.009598 |
| **1998-2003** | 12.56* | 8.78 | 16.86 | 0.0008 |
| **2003-2018** | 2.77* | 2.29 | 3.07 | 0.0016 |
| **2018-2021** | 10.05* | 7.01 | 11.69 | < 0.000001 |
| **2021-2025** | -7.84* | -9.38 | -6.41 | < 0.000001 |
| **Elderly Adults (65+)** | | | | |
| **1968-1984** | -4.78* | -5.74 | -3.96 | 0.011998 |
| **1984-1998** | 3.09* | 0.76 | 4.34 | 0.041192 |
| **1998-2005** | 8.86* | 3.63 | 14.31 | 0.009998 |
| **2005-2018** | 4.02* | 2.18 | 8.41 | 0.009198 |
| **2018-2021** | 8.35* | 4.64 | 9.96 | < 0.000001 |
| **2021-2025** | -6.52* | -8.21 | -5.02 | < 0.000001 |
| APC = annual percent change; * Indicates that the APC is significantly different from zero at α = 0.05. AAMR = age-adjusted mortality rate; CI = Confidence Interval | | | | |

| **Supplementary Table 5: Average Annual Percent Change (AAPC) of Obesity-Related Age-Adjusted Mortality Rates (AAMRs) per 100,000 in Adults Aged 25 and Older in the United States (1968–2025)** | | | | |
| --- | --- | --- | --- | --- |
| **Cohort** | **AAPC (1968-2025)** | **Lower 95% CI** | **Upper 95%**  **CI** | **P-value** |
| **Overall** | 1.84* | 1.72 | 1.98 | < 0.000001 |
| **Female** | 1.37* | 1.25 | 1.51 | < 0.000001 |
| **Male** | 2.41* | 2.27 | 2.59 | < 0.000001 |
| **Midwest** | 1.38* | 1.19 | 1.57 | < 0.000001 |
| **Northeast** | 2.30* | 2.09 | 2.52 | < 0.000001 |
| **South** | 1.95* | 1.81 | 2.1 | < 0.000001 |
| **West** | 1.93* | 1.74 | 2.2 | < 0.000001 |
| **Ages 25-44** | 2.58* | 2.37 | 2.87 | < 0.000001 |
| **Ages 45-64** | 1.97* | 1.83 | 2.14 | < 0.000001 |
| **Ages 65+** | 1.27* | 1.14 | 1.44 | < 0.000001 |
| **Black or African American** | 1.76* | 1.56 | 2 | < 0.000001 |
| **White** | 1.94* | 1.83 | 2.07 | < 0.000001 |
| **Races 1999-2025** | | | | |
| **American Indian or Alaska Native** | 3.91* | 2.76 | 5.12 | < 0.000001 |
| **Asian or Pacific Islander** | 4.30* | 3.38 | 5.31 | < 0.000001 |
| **Black or African American** | 2.21* | 1.91 | 2.48 | < 0.000001 |
| **White** | 3.26* | 3.07 | 3.46 | < 0.000001 |
| APC = annual percent change; * Indicates that the APC is significantly different from zero at α = 0.05. AAMR = age-adjusted mortality rate; CI = Confidence Interval | | | | |

| **Supplementary Table 6: Age-Stratified Obesity-Related Age-Adjusted Mortality Rates (AAMRs) per 100,000 in Adults Aged 25 and Older in the United States (1968–2025)** | | | | | | | | | |
| --- | --- | --- | --- | --- | --- | --- | --- | --- | --- |
| **Year** | **Young and Middle-aged Adults (25-44)** | | | **Old Adults (45-64)** | | | **Elderly (65+)** | | |
|  | **AAMR** | **Lower 95% CI** | **Upper 95% CI** | **AAMR** | **Lower 95% CI** | **Upper 95% CI** | **AAMR** | **Lower 95% CI** | **Upper 95% CI** |
| **1968** | 0.34 | 0.29 | 0.39 | 1.33 | 1.22 | 1.44 | 2.75 | 2.51 | 3 |
| **1969** | 0.32 | 0.27 | 0.37 | 1.28 | 1.18 | 1.39 | 2.19 | 1.98 | 2.4 |
| **1970** | 0.29 | 0.24 | 0.34 | 1.27 | 1.16 | 1.38 | 2.44 | 2.22 | 2.67 |
| **1971** | 0.39 | 0.33 | 0.45 | 1.12 | 1.02 | 1.22 | 2.32 | 2.11 | 2.54 |
| **1972** | 0.36 | 0.31 | 0.42 | 1.25 | 1.15 | 1.36 | 2.24 | 2.03 | 2.45 |
| **1973** | 0.39 | 0.33 | 0.45 | 1.26 | 1.16 | 1.37 | 2.27 | 2.06 | 2.48 |
| **1974** | 0.4 | 0.35 | 0.46 | 1.27 | 1.17 | 1.38 | 2.01 | 1.81 | 2.2 |
| **1975** | 0.35 | 0.3 | 0.41 | 1.02 | 0.93 | 1.12 | 1.93 | 1.75 | 2.12 |
| **1976** | 0.39 | 0.34 | 0.45 | 1.04 | 0.94 | 1.13 | 1.67 | 1.5 | 1.83 |
| **1977** | 0.39 | 0.33 | 0.44 | 1.02 | 0.93 | 1.12 | 1.74 | 1.57 | 1.91 |
| **1978** | 0.36 | 0.31 | 0.41 | 0.99 | 0.9 | 1.09 | 1.76 | 1.59 | 1.93 |
| **1979** | 0.37 | 0.32 | 0.42 | 0.95 | 0.86 | 1.04 | 1.44 | 1.29 | 1.59 |
| **1980** | 0.42 | 0.37 | 0.48 | 1.03 | 0.93 | 1.12 | 1.53 | 1.37 | 1.69 |
| **1981** | 0.4 | 0.35 | 0.46 | 0.89 | 0.8 | 0.98 | 1.35 | 1.21 | 1.49 |
| **1982** | 0.32 | 0.27 | 0.37 | 0.85 | 0.76 | 0.93 | 1.27 | 1.13 | 1.4 |
| **1983** | 0.31 | 0.27 | 0.36 | 0.89 | 0.81 | 0.98 | 1.35 | 1.21 | 1.49 |
| **1984** | 0.29 | 0.25 | 0.34 | 0.84 | 0.76 | 0.93 | 1.27 | 1.13 | 1.4 |
| **1985** | 0.33 | 0.29 | 0.38 | 0.94 | 0.85 | 1.03 | 1.22 | 1.09 | 1.34 |
| **1986** | 0.32 | 0.27 | 0.36 | 0.95 | 0.86 | 1.05 | 1.29 | 1.16 | 1.42 |
| **1987** | 0.32 | 0.28 | 0.36 | 0.93 | 0.84 | 1.02 | 1.18 | 1.06 | 1.3 |
| **1988** | 0.34 | 0.3 | 0.38 | 1.02 | 0.92 | 1.11 | 1.33 | 1.2 | 1.46 |
| **1989** | 0.37 | 0.33 | 0.42 | 1.21 | 1.11 | 1.32 | 1.57 | 1.43 | 1.71 |
| **1990** | 0.34 | 0.3 | 0.39 | 1.06 | 0.96 | 1.15 | 1.58 | 1.44 | 1.73 |
| **1991** | 0.45 | 0.4 | 0.49 | 1.18 | 1.08 | 1.28 | 1.39 | 1.26 | 1.52 |
| **1992** | 0.47 | 0.43 | 0.52 | 1.26 | 1.16 | 1.36 | 1.42 | 1.29 | 1.55 |
| **1993** | 0.49 | 0.44 | 0.54 | 1.38 | 1.28 | 1.48 | 1.59 | 1.46 | 1.73 |
| **1994** | 0.52 | 0.47 | 0.57 | 1.39 | 1.29 | 1.49 | 1.65 | 1.51 | 1.79 |
| **1995** | 0.55 | 0.5 | 0.6 | 1.41 | 1.31 | 1.51 | 1.81 | 1.66 | 1.95 |
| **1996** | 0.54 | 0.49 | 0.59 | 1.65 | 1.54 | 1.75 | 1.76 | 1.62 | 1.91 |
| **1997** | 0.63 | 0.57 | 0.68 | 1.64 | 1.53 | 1.74 | 1.83 | 1.69 | 1.98 |
| **1998** | 0.7 | 0.64 | 0.76 | 1.74 | 1.64 | 1.85 | 1.78 | 1.64 | 1.93 |
| **1999** | 0.83 | 0.76 | 0.89 | 1.86 | 1.75 | 1.97 | 2.12 | 1.97 | 2.27 |
| **2000** | 0.88 | 0.82 | 0.94 | 2.26 | 2.14 | 2.38 | 2.24 | 2.09 | 2.4 |
| **2001** | 0.93 | 0.86 | 0.99 | 2.34 | 2.22 | 2.45 | 2.26 | 2.11 | 2.42 |
| **2002** | 1.14 | 1.06 | 1.21 | 2.71 | 2.59 | 2.84 | 2.49 | 2.32 | 2.65 |
| **2003** | 1.08 | 1.01 | 1.15 | 2.81 | 2.69 | 2.94 | 2.97 | 2.79 | 3.15 |
| **2004** | 1.08 | 1.01 | 1.15 | 2.91 | 2.79 | 3.04 | 3.2 | 3.02 | 3.39 |
| **2005** | 1.18 | 1.11 | 1.26 | 3.17 | 3.04 | 3.3 | 3.4 | 3.21 | 3.59 |
| **2006** | 1.14 | 1.06 | 1.21 | 3.19 | 3.07 | 3.32 | 3.51 | 3.32 | 3.7 |
| **2007** | 1.24 | 1.16 | 1.31 | 3.23 | 3.1 | 3.36 | 3.5 | 3.31 | 3.69 |
| **2008** | 1.29 | 1.21 | 1.37 | 3.17 | 3.05 | 3.3 | 3.6 | 3.41 | 3.79 |
| **2009** | 1.29 | 1.21 | 1.37 | 3.35 | 3.22 | 3.48 | 3.9 | 3.7 | 4.09 |
| **2010** | 1.35 | 1.26 | 1.43 | 3.35 | 3.23 | 3.48 | 3.99 | 3.8 | 4.19 |
| **2011** | 1.39 | 1.31 | 1.47 | 3.53 | 3.4 | 3.66 | 4.36 | 4.16 | 4.57 |
| **2012** | 1.35 | 1.26 | 1.43 | 3.65 | 3.52 | 3.78 | 4.52 | 4.32 | 4.73 |
| **2013** | 1.35 | 1.27 | 1.43 | 3.73 | 3.6 | 3.86 | 4.73 | 4.53 | 4.94 |
| **2014** | 1.61 | 1.52 | 1.7 | 3.75 | 3.62 | 3.88 | 4.94 | 4.73 | 5.14 |
| **2015** | 1.61 | 1.52 | 1.7 | 4.13 | 3.99 | 4.27 | 5.29 | 5.08 | 5.5 |
| **2016** | 1.66 | 1.57 | 1.75 | 4.31 | 4.17 | 4.45 | 5.36 | 5.15 | 5.57 |
| **2017** | 1.65 | 1.57 | 1.74 | 4.13 | 3.99 | 4.27 | 5.32 | 5.12 | 5.52 |
| **2018** | 1.56 | 1.48 | 1.65 | 4.15 | 4.01 | 4.29 | 5.59 | 5.38 | 5.79 |
| **2019** | 1.61 | 1.52 | 1.7 | 4.43 | 4.28 | 4.57 | 5.74 | 5.54 | 5.95 |
| **2020** | 2.13 | 2.03 | 2.23 | 5.43 | 5.27 | 5.59 | 6.57 | 6.36 | 6.79 |
| **2021** | 2.38 | 2.28 | 2.49 | 5.85 | 5.68 | 6.02 | 7.31 | 7.09 | 7.54 |
| **2022** | 2.12 | 2.02 | 2.22 | 5.07 | 4.91 | 5.22 | 6.92 | 6.71 | 7.14 |
| **2023** | 1.92 | 1.83 | 2.01 | 4.71 | 4.56 | 4.86 | 6 | 5.8 | 6.2 |
| **2024** | 1.76 | 1.68 | 1.85 | 4.41 | 4.27 | 4.56 | 5.79 | 5.6 | 5.98 |
| **2025** | 1.65 | 1.57 | 1.74 | 4.27 | 4.13 | 4.42 | 5.6 | 5.41 | 5.79 |
| AAMR = Age-Adjusted Mortality Rate; CI = Confidence Interval | | | | | | | | | |

| **Supplementary Table 7: Race-Stratified Obesity-Related Age-Adjusted Mortality Rates (AAMRs) per 100,000 in Adults Aged 25 and Older in the United States (1968–2025)** | | | | | | |
| --- | --- | --- | --- | --- | --- | --- |
| **Year** | **Blacks or African Americans** | | | **Whites** | | |
|  | **AAMR** | **Lower 95% CI** | **Upper 95% CI** | **AAMR** | **Lower 95% CI** | **Upper 95% CI** |
| **1968** | 1.8 | 1.53 | 2.07 | 1.08 | 1.02 | 1.15 |
| **1969** | 1.88 | 1.6 | 2.16 | 0.93 | 0.87 | 0.99 |
| **1970** | 1.71 | 1.45 | 1.98 | 0.98 | 0.92 | 1.05 |
| **1971** | 1.56 | 1.31 | 1.8 | 0.96 | 0.9 | 1.02 |
| **1972** | 2.17 | 1.87 | 2.46 | 0.92 | 0.86 | 0.98 |
| **1973** | 1.69 | 1.44 | 1.94 | 0.99 | 0.93 | 1.06 |
| **1974** | 1.67 | 1.42 | 1.92 | 0.95 | 0.89 | 1.01 |
| **1975** | 1.53 | 1.29 | 1.76 | 0.83 | 0.77 | 0.88 |
| **1976** | 1.65 | 1.41 | 1.89 | 0.79 | 0.73 | 0.84 |
| **1977** | 1.62 | 1.38 | 1.86 | 0.79 | 0.74 | 0.85 |
| **1978** | 1.57 | 1.34 | 1.79 | 0.78 | 0.72 | 0.83 |
| **1979** | 1.43 | 1.21 | 1.64 | 0.72 | 0.67 | 0.77 |
| **1980** | 1.79 | 1.54 | 2.03 | 0.76 | 0.71 | 0.81 |
| **1981** | 1.49 | 1.27 | 1.71 | 0.69 | 0.64 | 0.73 |
| **1982** | 1.42 | 1.2 | 1.63 | 0.62 | 0.57 | 0.67 |
| **1983** | 1.47 | 1.26 | 1.68 | 0.63 | 0.59 | 0.68 |
| **1984** | 1.51 | 1.29 | 1.72 | 0.59 | 0.54 | 0.63 |
| **1985** | 1.5 | 1.29 | 1.71 | 0.64 | 0.6 | 0.69 |
| **1986** | 1.64 | 1.43 | 1.86 | 0.64 | 0.59 | 0.68 |
| **1987** | 1.64 | 1.42 | 1.86 | 0.61 | 0.57 | 0.66 |
| **1988** | 1.84 | 1.61 | 2.06 | 0.66 | 0.62 | 0.71 |
| **1989** | 2.02 | 1.79 | 2.26 | 0.78 | 0.74 | 0.83 |
| **1990** | 1.89 | 1.66 | 2.12 | 0.73 | 0.68 | 0.78 |
| **1991** | 1.92 | 1.7 | 2.15 | 0.78 | 0.74 | 0.83 |
| **1992** | 2.09 | 1.86 | 2.32 | 0.82 | 0.77 | 0.87 |
| **1993** | 2.29 | 2.05 | 2.52 | 0.89 | 0.84 | 0.94 |
| **1994** | 2.24 | 2.01 | 2.47 | 0.93 | 0.88 | 0.98 |
| **1995** | 2.21 | 1.99 | 2.44 | 0.99 | 0.94 | 1.04 |
| **1996** | 2.36 | 2.13 | 2.6 | 1.05 | 1 | 1.1 |
| **1997** | 2.51 | 2.28 | 2.75 | 1.09 | 1.04 | 1.14 |
| **1998** | 2.6 | 2.36 | 2.83 | 1.15 | 1.09 | 1.2 |
| **1999** | 2.57 | 2.34 | 2.81 | 1.35 | 1.29 | 1.41 |
| **2000** | 2.94 | 2.7 | 3.19 | 1.5 | 1.44 | 1.56 |
| **2001** | 3.12 | 2.87 | 3.37 | 1.55 | 1.49 | 1.61 |
| **2002** | 3.47 | 3.21 | 3.72 | 1.81 | 1.75 | 1.88 |
| **2003** | 3.21 | 2.97 | 3.46 | 2.01 | 1.94 | 2.08 |
| **2004** | 3.91 | 3.64 | 4.18 | 1.99 | 1.92 | 2.06 |
| **2005** | 3.95 | 3.69 | 4.22 | 2.17 | 2.09 | 2.24 |
| **2006** | 3.83 | 3.56 | 4.09 | 2.2 | 2.13 | 2.27 |
| **2007** | 3.72 | 3.46 | 3.97 | 2.26 | 2.19 | 2.33 |
| **2008** | 3.85 | 3.6 | 4.11 | 2.25 | 2.18 | 2.33 |
| **2009** | 3.91 | 3.65 | 4.17 | 2.43 | 2.35 | 2.5 |
| **2010** | 3.96 | 3.7 | 4.21 | 2.46 | 2.39 | 2.54 |
| **2011** | 4.33 | 4.07 | 4.6 | 2.61 | 2.53 | 2.69 |
| **2012** | 4.25 | 3.99 | 4.51 | 2.67 | 2.59 | 2.75 |
| **2013** | 4.29 | 4.03 | 4.55 | 2.76 | 2.68 | 2.84 |
| **2014** | 4.88 | 4.61 | 5.16 | 2.87 | 2.79 | 2.94 |
| **2015** | 5.11 | 4.83 | 5.38 | 3.04 | 2.96 | 3.12 |
| **2016** | 5.18 | 4.91 | 5.45 | 3.14 | 3.06 | 3.22 |
| **2017** | 4.87 | 4.61 | 5.13 | 3.13 | 3.05 | 3.21 |
| **2018** | 5.02 | 4.76 | 5.29 | 3.16 | 3.08 | 3.24 |
| **2019** | 5.34 | 5.07 | 5.61 | 3.29 | 3.21 | 3.38 |
| **2020** | 6.7 | 6.4 | 7 | 3.97 | 3.88 | 4.06 |
| **2021** | 7.56 | 7.24 | 7.88 | 4.37 | 4.27 | 4.46 |
| **2022** | 6.64 | 6.34 | 6.94 | 3.91 | 3.82 | 4 |
| **2023** | 5.92 | 5.64 | 6.2 | 3.57 | 3.49 | 3.66 |
| **2024** | 5.28 | 5.02 | 5.55 | 3.41 | 3.32 | 3.49 |
| **2025** | 5.31 | 5.05 | 5.58 | 3.28 | 3.19 | 3.36 |
| AAMR = Age-Adjusted Mortality Rate; CI = Confidence Interval | | | | | | |

| **Supplementary Table 8: Other Races-Stratified Obesity-Related Age-Adjusted Mortality Rates (AAMRs) per 100,000 in Adults Aged 25 and Older in the United States (1999–2025)** | | | | | | |
| --- | --- | --- | --- | --- | --- | --- |
| **Year** | **American Indian or Alaska Native** | | | **Asian or Pacific Islander** | | |
|  | **AAMR** | **Lower 95% CI** | **Upper 95% CI** | **AAMR** | **Lower 95% CI** | **Upper 95% CI** |
| **1999** | 6.5 | 5.01 | 8.31 | 1 | 0.76 | 1.29 |
| **2000** | 8.09 | 6.41 | 9.78 | 1.31 | 1.03 | 1.65 |
| **2001** | 7.25 | 5.83 | 8.66 | 0.93 | 0.72 | 1.18 |
| **2002** | 9.18 | 7.46 | 10.89 | 1.47 | 1.18 | 1.77 |
| **2003** | 10.76 | 8.97 | 12.54 | 1.63 | 1.33 | 1.93 |
| **2004** | 9.4 | 7.79 | 11.01 | 1.59 | 1.31 | 1.88 |
| **2005** | 12.54 | 10.78 | 14.29 | 1.92 | 1.61 | 2.23 |
| **2006** | 12.06 | 10.34 | 13.78 | 1.95 | 1.65 | 2.25 |
| **2007** | 11.8 | 10.19 | 13.41 | 2.03 | 1.73 | 2.33 |
| **2008** | 13.66 | 11.95 | 15.36 | 2.32 | 2.01 | 2.64 |
| **2009** | 16.62 | 14.73 | 18.51 | 2.45 | 2.13 | 2.76 |
| **2010** | 16.73 | 14.92 | 18.54 | 2.38 | 2.08 | 2.68 |
| **2011** | 14.5 | 12.87 | 16.14 | 2.53 | 2.23 | 2.84 |
| **2012** | 16.65 | 14.9 | 18.39 | 2.64 | 2.33 | 2.94 |
| **2013** | 16.4 | 14.71 | 18.09 | 2.54 | 2.25 | 2.84 |
| **2014** | 19.51 | 17.67 | 21.35 | 2.9 | 2.59 | 3.2 |
| **2015** | 18.19 | 16.47 | 19.92 | 3.58 | 3.24 | 3.91 |
| **2016** | 21.6 | 19.74 | 23.47 | 3.19 | 2.88 | 3.5 |
| **2017** | 20.81 | 19.03 | 22.6 | 3.39 | 3.08 | 3.71 |
| **2018** | 21.06 | 19.3 | 22.82 | 3.64 | 3.33 | 3.96 |
| **2019** | 20.44 | 18.71 | 22.17 | 3.77 | 3.45 | 4.08 |
| **2020** | 43.79 | 41.3 | 46.27 | 7.08 | 6.65 | 7.5 |
| **2021** | 53.99 | 51.17 | 56.95 | 7.77 | 7.32 | 8.23 |
| **2022** | 38.23 | 35.86 | 40.73 | 5.4 | 5.04 | 5.78 |
| **2023** | 30.82 | 28.73 | 33.04 | 4.64 | 4.3 | 4.99 |
| **2024** | 26.68 | 24.8 | 28.69 | 4.37 | 4.06 | 4.7 |
| **2025** | 25.59 | 23.74 | 27.55 | 4.24 | 3.93 | 4.56 |
| AAMR = Age-Adjusted Mortality Rate; CI = Confidence Interval | | | | | | |

| **Supplementary Table 9: Obesity-Related Age-Adjusted Mortality Rates (AAMRs) per 100,000, Stratified by Census Region (CR), in Adults Aged 25 and Older in the United States (1968–2025)** | | | | |
| --- | --- | --- | --- | --- |
| **Year** | **CR: Northeast** | **CR: Midwest** | **CR: South** | **CR: West** |
|  | **AAMR** | **AAMR** | **AAMR** | **AAMR** |
| **1968** | 0.75 | 1.5 | 1.17 | 1.14 |
| **1969** | 0.67 | 1.32 | 1.11 | 0.89 |
| **1970** | 0.76 | 1.35 | 1.07 | 0.96 |
| **1971** | 0.83 | 1.3 | 0.96 | 0.94 |
| **1972** | 0.69 | 1.32 | 1.13 | 0.91 |
| **1973** | 0.73 | 1.26 | 1.13 | 1.09 |
| **1974** | 0.75 | 1.3 | 1.02 | 0.92 |
| **1975** | 0.63 | 1.13 | 0.91 | 0.86 |
| **1976** | 0.58 | 1.04 | 0.98 | 0.78 |
| **1977** | 0.6 | 1.05 | 0.98 | 0.78 |
| **1978** | 0.63 | 1.1 | 0.92 | 0.66 |
| **1979** | 0.62 | 0.94 | 0.84 | 0.63 |
| **1980** | 0.7 | 1 | 0.87 | 0.77 |
| **1981** | 0.62 | 0.81 | 0.84 | 0.7 |
| **1982** | 0.59 | 0.85 | 0.68 | 0.6 |
| **1983** | 0.6 | 0.78 | 0.78 | 0.64 |
| **1984** | 0.56 | 0.71 | 0.74 | 0.62 |
| **1985** | 0.75 | 0.76 | 0.75 | 0.53 |
| **1986** | 0.66 | 0.79 | 0.78 | 0.62 |
| **1987** | 0.7 | 0.69 | 0.76 | 0.59 |
| **1988** | 0.72 | 0.81 | 0.86 | 0.61 |
| **1989** | 0.83 | 0.98 | 1.01 | 0.65 |
| **1990** | 0.78 | 0.89 | 0.98 | 0.56 |
| **1991** | 0.76 | 1 | 1.02 | 0.62 |
| **1992** | 0.82 | 0.93 | 1.1 | 0.74 |
| **1993** | 0.91 | 0.98 | 1.24 | 0.77 |
| **1994** | 0.97 | 1.03 | 1.18 | 0.89 |
| **1995** | 0.89 | 1.05 | 1.29 | 0.99 |
| **1996** | 1 | 1.08 | 1.38 | 1.04 |
| **1997** | 1.09 | 1.11 | 1.37 | 1.16 |
| **1998** | 1.18 | 1.23 | 1.49 | 1.04 |
| **1999** | 1.35 | 1.43 | 1.56 | 1.29 |
| **2000** | 1.52 | 1.46 | 1.88 | 1.5 |
| **2001** | 1.49 | 1.66 | 1.88 | 1.53 |
| **2002** | 1.63 | 2 | 2.13 | 1.77 |
| **2003** | 1.84 | 1.99 | 2.35 | 1.9 |
| **2004** | 1.89 | 1.96 | 2.51 | 1.92 |
| **2005** | 1.99 | 2.2 | 2.62 | 2.16 |
| **2006** | 2.06 | 2.16 | 2.56 | 2.29 |
| **2007** | 2.07 | 2.26 | 2.55 | 2.28 |
| **2008** | 2.05 | 2.25 | 2.56 | 2.48 |
| **2009** | 2.17 | 2.45 | 2.73 | 2.5 |
| **2010** | 2.26 | 2.53 | 2.68 | 2.58 |
| **2011** | 2.57 | 2.59 | 2.85 | 2.65 |
| **2012** | 2.55 | 2.75 | 2.91 | 2.62 |
| **2013** | 2.59 | 3.05 | 2.92 | 2.67 |
| **2014** | 2.68 | 3.27 | 3.17 | 2.77 |
| **2015** | 2.99 | 3.37 | 3.26 | 3.01 |
| **2016** | 3.06 | 3.45 | 3.37 | 3.07 |
| **2017** | 2.92 | 3.38 | 3.3 | 3.16 |
| **2018** | 2.99 | 3.4 | 3.38 | 3.05 |
| **2019** | 3.16 | 3.33 | 3.77 | 3.02 |
| **2020** | 3.69 | 4.09 | 4.47 | 3.93 |
| **2021** | 3.69 | 4.33 | 5.04 | 4.52 |
| **2022** | 3.5 | 3.92 | 4.34 | 4.14 |
| **2023** | 3.1 | 3.58 | 4 | 3.63 |
| **2024** | 2.88 | 3.33 | 3.79 | 3.41 |
| **2025** | 2.81 | 3.38 | 3.62 | 3.2 |

| **Supplementary Table 10: Obesity-Related Age-Adjusted Mortality Rates (AAMRs) per 100,000, Stratified by State, in Adults Aged 25 and Older in the United States (1968–1978)** | | | | |
| --- | --- | --- | --- | --- |
| **State** | **Rank** | **Deaths** | **Age Adjusted Mortality Rate** | **Percentile** |
| **New Hampshire** | 1 | 87 | 1.87 | 98 |
| **Kansas** | 2 | 238 | 1.6 | 96 |
| **Wisconsin** | 3 | 435 | 1.55 | 94 |
| **Idaho** | 4 | 66 | 1.53 | 92 |
| **Indiana** | 5 | 478 | 1.52 | 90 |
| **Michigan** | 6 | 735 | 1.47 | 88 |
| **Kentucky** | 7 | 293 | 1.45 | 86 |
| **New Mexico** | 8 | 77 | 1.38 | 84 |
| **South Dakota** | 9 | 60 | 1.36 | 82 |
| **Nebraska** | 10 | 129 | 1.33 | 80 |
| **Louisiana** | 11 | 271 | 1.28 | 78 |
| **Oklahoma** | 12 | 220 | 1.24 | 76 |
| **North Carolina** | 13.5 | 388 | 1.23 | 73 |
| **Washington** | 13.5 | 252 | 1.23 | 73 |
| **Georgia** | 15.5 | 325 | 1.22 | 69 |
| **South Carolina** | 15.5 | 177 | 1.22 | 69 |
| **Ohio** | 17 | 756 | 1.2 | 67 |
| **Minnesota** | 18 | 277 | 1.17 | 65 |
| **Nevada** | 19 | 34 | 1.15 | 63 |
| **Iowa** | 20 | 220 | 1.14 | 61 |
| **Arkansas** | 22.5 | 146 | 1.12 | 53 |
| **Missouri** | 22.5 | 351 | 1.12 | 53 |
| **West Virginia** | 22.5 | 132 | 1.12 | 53 |
| **Wyoming** | 22.5 | 24 | 1.12 | 53 |
| **Utah** | 25 | 62 | 1.1 | 51 |
| **Montana** | 26 | 45 | 1.07 | 50 |
| **Alabama** | 27 | 227 | 1.06 | 48 |
| **Maine** | 29 | 68 | 1.05 | 42 |
| **Maryland** | 29 | 239 | 1.05 | 42 |
| **Mississippi** | 29 | 141 | 1.05 | 42 |
| **Tennessee** | 31 | 248 | 0.99 | 40 |
| **Vermont** | 32 | 28 | 0.98 | 38 |
| **Alaska** | 33.5 | 12 | 0.97 | 34 |
| **North Dakota** | 33.5 | 38 | 0.97 | 34 |
| **Oregon** | 35 | 133 | 0.94 | 32 |
| **Texas** | 36 | 599 | 0.9 | 30 |
| **Virginia** | 37 | 238 | 0.88 | 28 |
| **Illinois** | 38 | 589 | 0.87 | 26 |
| **Delaware** | 39 | 28 | 0.84 | 25 |
| **Rhode Island** | 40 | 53 | 0.81 | 23 |
| **California** | 41 | 971 | 0.79 | 21 |
| **Arizona** | 42.5 | 95 | 0.78 | 17 |
| **Pennsylvania** | 42.5 | 616 | 0.78 | 17 |
| **District of Columbia** | 44 | 33 | 0.76 | 15 |
| **Florida** | 45 | 391 | 0.71 | 13 |
| **Colorado** | 46 | 93 | 0.69 | 11 |
| **New York** | 47 | 781 | 0.66 | 9 |
| **Connecticut** | 49 | 118 | 0.6 | 3 |
| **Hawaii** | 49 | 27 | 0.6 | 3 |
| **New Jersey** | 49 | 277 | 0.6 | 3 |
| **Massachusetts** | 51 | 183 | 0.49 | 1 |

| **Supplementary Table 11: Obesity-Related Age-Adjusted Mortality Rates (AAMRs) per 100,000, Stratified by State, in Adults Aged 25 and Older in the United States (2018–2025)** | | | | |
| --- | --- | --- | --- | --- |
| **State** | **Rank** | **Deaths** | **Age Adjusted Mortality Rate** | **Percentile** |
| **West Virginia** | 1 | 781 | 7.11 | 98 |
| **North Dakota** | 2 | 304 | 6.76 | 96 |
| **New Mexico** | 3.5 | 733 | 5.96 | 92 |
| **Vermont** | 3.5 | 255 | 5.96 | 92 |
| **Maine** | 5 | 541 | 5.42 | 90 |
| **Arkansas** | 6 | 906 | 5.13 | 88 |
| **Delaware** | 7 | 313 | 4.92 | 86 |
| **Montana** | 8 | 344 | 4.88 | 84 |
| **Alaska** | 9 | 196 | 4.84 | 82 |
| **Kentucky** | 10 | 1261 | 4.82 | 80 |
| **Kansas** | 11 | 823 | 4.78 | 78 |
| **North Carolina** | 12 | 3021 | 4.76 | 76 |
| **Utah** | 13 | 767 | 4.71 | 75 |
| **Tennessee** | 14 | 1951 | 4.7 | 73 |
| **Louisiana** | 15.5 | 1216 | 4.62 | 69 |
| **New Hampshire** | 15.5 | 429 | 4.62 | 69 |
| **Idaho** | 17.5 | 526 | 4.59 | 65 |
| **Oregon** | 17.5 | 1263 | 4.59 | 65 |
| **Nebraska** | 19 | 528 | 4.53 | 63 |
| **South Carolina** | 20 | 1411 | 4.33 | 61 |
| **Alabama** | 21 | 1308 | 4.32 | 59 |
| **Rhode Island** | 22 | 291 | 4.28 | 57 |
| **Mississippi** | 23 | 703 | 4.25 | 55 |
| **Indiana** | 24 | 1726 | 4.24 | 53 |
| **Arizona** | 25 | 1854 | 4.18 | 51 |
| **Wisconsin** | 26 | 1601 | 4.16 | 50 |
| **Georgia** | 27 | 2445 | 4 | 48 |
| **Texas** | 28 | 6383 | 3.99 | 46 |
| **District of Columbia** | 29.5 | 139 | 3.89 | 42 |
| **Iowa** | 29.5 | 772 | 3.89 | 42 |
| **Nevada** | 31 | 734 | 3.85 | 40 |
| **Colorado** | 32 | 1314 | 3.84 | 38 |
| **Michigan** | 33 | 2353 | 3.83 | 36 |
| **Washington** | 34 | 1735 | 3.65 | 34 |
| **Ohio** | 35 | 2603 | 3.63 | 32 |
| **South Dakota** | 36 | 199 | 3.57 | 30 |
| **Minnesota** | 37 | 1282 | 3.55 | 28 |
| **Florida** | 38 | 5111 | 3.45 | 26 |
| **Oklahoma** | 40 | 757 | 3.36 | 21 |
| **Virginia** | 40 | 1754 | 3.36 | 21 |
| **Wyoming** | 40 | 119 | 3.36 | 21 |
| **Pennsylvania** | 42 | 2723 | 3.31 | 19 |
| **Missouri** | 43 | 1198 | 3.28 | 17 |
| **New York** | 44 | 3851 | 3.19 | 15 |
| **California** | 45 | 6937 | 3.09 | 13 |
| **Maryland** | 46 | 1134 | 3 | 11 |
| **Connecticut** | 47 | 687 | 2.98 | 9 |
| **New Jersey** | 48 | 1635 | 2.89 | 7 |
| **Illinois** | 49 | 2037 | 2.71 | 5 |
| **Massachusetts** | 50 | 1132 | 2.58 | 3 |
| **Hawaii** | 51 | 163 | 1.88 | 1 |
